# 1-{(*E*)-[4-(4-Hy­droxy­phen­yl)butan-2-yl­idene]amino}-3-phenyl­thio­urea: crystal structure, Hirshfeld surface analysis and computational study

**DOI:** 10.1107/S2056989021006666

**Published:** 2021-07-13

**Authors:** Ming Yueh Tan, Huey Chong Kwong, Karen A. Crouse, Thahira B. S. A. Ravoof, Edward R. T. Tiekink

**Affiliations:** a Department of Physical Science, Faculty of Applied Sciences, Tunku Abdul Rahman University College, 50932 Setapak, Kuala Lumpur, Malaysia; b Research Centre for Crystalline Materials, School of Medical and Life Sciences, Sunway University, 47500 Bandar Sunway, Selangor Darul Ehsan, Malaysia; c Department of Chemistry, Faculty of Science, Universiti Putra Malaysia, UPM, Serdang 43400, Malaysia; d Department of Chemistry, St. Francis Xavier University, PO Box 5000, Antigonish, NS B2G 2W5, Canada

**Keywords:** crystal structure, Schiff base, thio­urea, hydrogen bonding, Hirshfeld surface analysis

## Abstract

The title thio­urea derivative, C_17_H_19_N_3_OS, adopts a U-shaped conformation with the dihedral angle between the terminal aromatic rings being 73.64 (5)°. The major twist in the mol­ecule occurs about the ethane bond with the C_i_—C_e_—C_e_—C_b_ torsion angle being −78.12 (18)°; i = imine, e = ethane and b = benzene. The configuration about the imine bond is *E*, the N-bound H atoms lie on opposite sides of the mol­ecule and an intra­molecular amine-N—H⋯N(imine) hydrogen bond is evident. In the mol­ecular packing, hydroxyl-O—H⋯S(thione) and amine-N—H⋯O hydrogen bonding feature within a linear, supra­molecular chain. The chains are connected into a layer in the *ab* plane by a combination of methyl­ene-C—H⋯S(thione), methyl­ene-C—H⋯O(hydrox­yl), methyl-C—H⋯π(phen­yl) and phenyl-C—H⋯π(hy­droxy­benzene) inter­actions. The layers stack without directional inter­actions between them. The analysis of the calculated Hirshfeld surface highlights the presence of weak methyl-C—H⋯O(hydrox­yl) and H⋯H inter­actions in the inter-layer region. Computational chemistry indicates that dispersion energy is the major contributor to the overall stabilization of the mol­ecular packing.

## Abstract 2

The title thio­urea derivative adopts a U-shaped conformation, which incorporates an intra­molecular amine-N—H⋯N(imine) hydrogen bond. In the mol­ecular packing, supra­molecular chains are formed through hydroxyl-O—H⋯S(thione) and amine-N—H⋯O hydrogen bonding.

## Chemical context   

Raspberry ketone, also known as 4-(4-hy­droxy­phen­yl)-2-butanone (C_10_H_12_O_2_), is a natural phenolic compound found in raspberries, kiwi fruit, brewed coffee, yew and orchid flowers (Lee, 2016 ▸). This ketone is the primary compound responsible for the fruity aroma and has long been used commercially as a fragrance and flavouring agent for cosmetics, perfume, food and beverages. The pharmaceutical attributes exhibited by this ketone include anti-androgenic activity in human breast cancer cells, de-pigmentation, anti-inflammatory activity and cardioprotective action in rats (Dziduch *et al.*, 2020 ▸; Yuan *et al.*, 2020 ▸). In this work, raspberry ketone was condensed with 4-phenyl-3-thio­semicarbazide to form the title thio­urea derivative, C_17_H_19_N_3_OS, hereafter designated as (I). Such compounds are of much inter­est due to their attractive and widespread pharmacological activities including anti-bacterial, anti-fungal, anti-tubercular, anti-convulsant, anti-tumour, anti-oxidant, anti-malarial and anti-helmintic properties (Dincel & Guzeldemirci, 2020 ▸). In a continuation of on-going studies on related derivatives and complexes (Tan, Ho *et al.* 2020 ▸; Tan, Kwong *et al.* 2020*a* ▸,*b* ▸), herein the synthesis, structure determination, Hirshfeld surface analysis and computational chemistry of (I) are reported. 
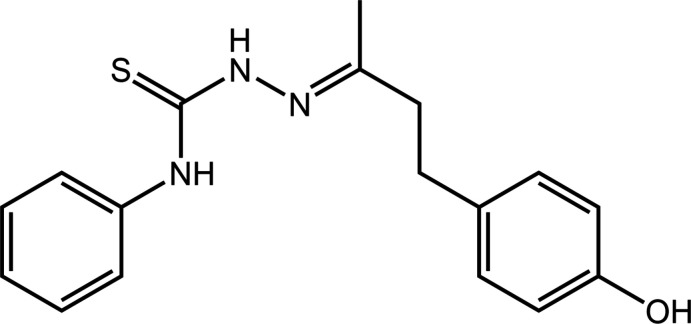


## Structural commentary   

**Figure 1:**
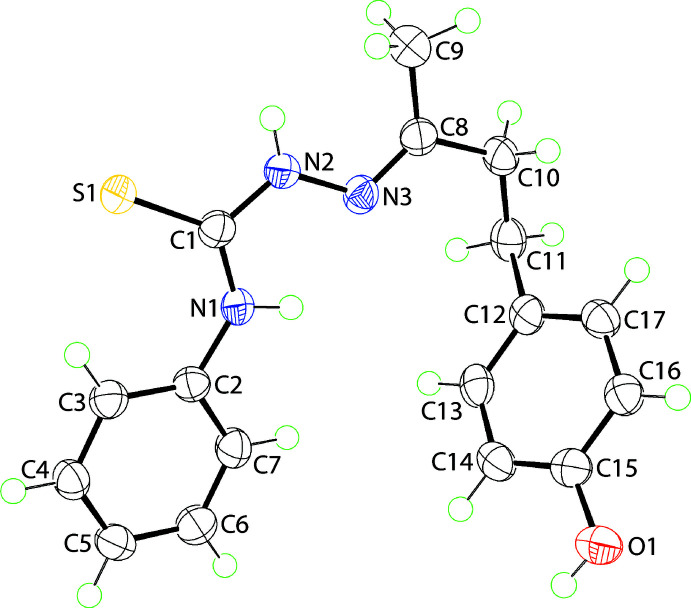
The mol­ecular structure of (I) showing the atom-labelling scheme and displacement ellipsoids at the 70% probability level.

The mol­ecular structure of (I), Fig. 1 ▸, comprises an almost planar central chromophore with the r.m.s. deviation for the C1, N1–N3 and S1 atoms being 0.0039 Å; the maximum deviation from the least-squares plane is 0.0054 (12) Å for the C1 atom. The pendant C2 and C8 atoms lie 0.065 (3) and 0.072 (2) Å out of and to the same side of the central plane. While the N1-bound phenyl ring is approximately co-planar with the central residue, forming a dihedral angle of 7.94 (8)°, the terminal 4-hy­droxy­benzene ring is not, forming a dihedral angle of 67.00 (4)°; the dihedral angle between the rings is 73.64 (5)°. This conformation arises as there is a twist about the ethane bond, *i.e*. the C8—C10—C11—C12 torsion angle is −78.12 (18)°. Globally, both aromatic residues lie to the same side of the mol­ecule so that it has a U-shaped conformation.

The C1—S1 bond length is 1.6910 (15) Å, the C1—N1 bond [1.340 (2) Å] is marginally shorter than the C1—N2 [1.356 (2) Å] bond, the formally C8—N3 double bond is 1.284 (2) Å and N2—N3 is 1.3857 (18) Å. These values, coupled with the observed planarity in this region of the mol­ecule, is suggestive of some delocalization of π-electron density over this residue. The configuration about the C8=N3 imine bond is *E*. The N-bound H atoms lie to opposite sides of the mol­ecule, a conformation that allows for the formation of an intra­molecular amine-N—H⋯N(imine) hydrogen bond, Table 1 ▸.

## Supra­molecular features   

**Figure 2:**
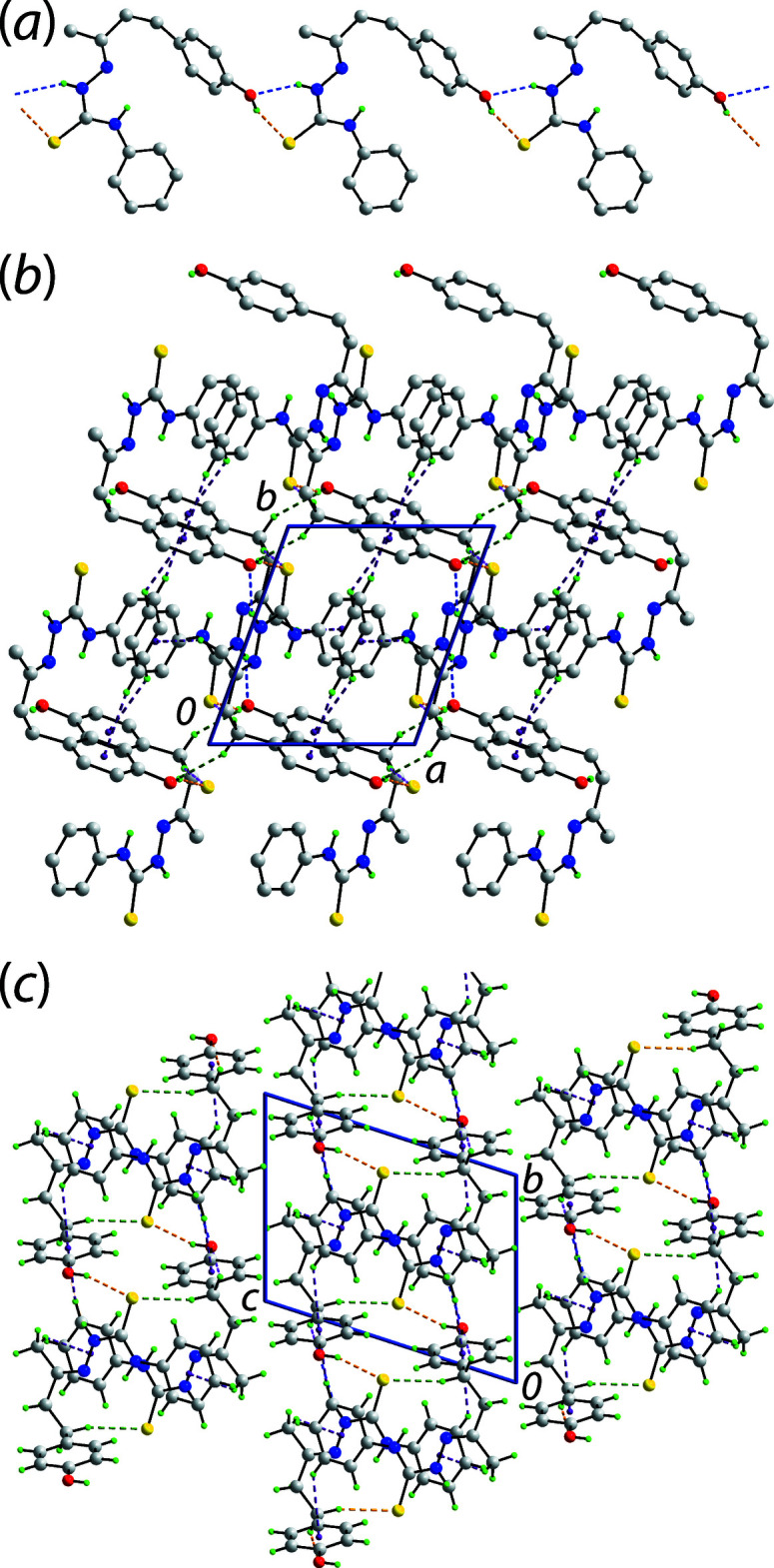
Mol­ecular packing in (I): (*a*) the supra­molecular chain sustained by hy­droxy-O—H⋯S(thione) and amine-N—H⋯O(hydrox­yl) hydrogen bonding shown as orange and blue dashed lines, respectively (non-participating H atoms omitted), (*b*) the supra­molecular layer whereby the chains of (*a*) are connected by methyl­ene-C—H⋯O(hy­droxy) (pink dashed lines), methyl­ene-C—H⋯O(thione) (green) and C—H⋯π (purple) inter­actions (non-participating H atoms omitted) and (*c*) a view of the unit-cell contents shown in projection down the *a* axis highlighting the stacking of layers.

In the crystal, hydrogen bonding leads to the formation of a linear, supra­molecular chain parallel to [ 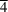 73]. These chains arise because the hydroxyl-O—H atom forms a hydrogen bond to the thione-S1 atom and the hydroxyl-O1 atom simultaneously accepts a N—H⋯O hydrogen bond from the amine-N2—H atom, Fig. 2 ▸(*a*). They are connected into a supra­molecular layer parallel to the *c* axis *via* methyl­ene-C—H⋯S(thione) and methyl­ene-C—H⋯O(hydrox­yl) inter­actions as well as methyl-C—H⋯π(phen­yl) and phenyl-C—H⋯π(hy­droxy­benzene) contacts, Table 1 ▸ and Fig. 2 ▸(*b*). The layers thus formed are two mol­ecules thick and stack along the *c*-axis direction without directional inter­actions between them, Fig. 2 ▸(*c*). Finally, as indicated in Fig. 2 ▸(*b*) and (*c*), the supra­molecular connectivity brings two sulfur atoms into close proximity, with an S1⋯S1^i^ separation of 3.3534 (6) Å, *cf.* the sum of the van der Waals radii of 3.60 Å (Spek, 2020 ▸); symmetry operation (i): 2 − *x*, −*y*, 1 − *z*.

## Analysis of the Hirshfeld surfaces   

The Hirshfeld surface analysis comprising the calculation of the *d*_norm_ surface (McKinnon *et al.*, 2004 ▸), electrostatic potential (Spackman *et al.*, 2008 ▸), using the wave function at the HF/STO-3G level of theory, and two-dimensional fingerprint plots (Spackman & McKinnon, 2002 ▸) were generated to further elucidate the inter­actions in the crystal of (I), in particular within the inter-layer region. This study was carried out using *Crystal Explorer 17* (Turner *et al.*, 2017 ▸) following literature procedures (Tan *et al.*, 2019 ▸).

**Figure 3:**
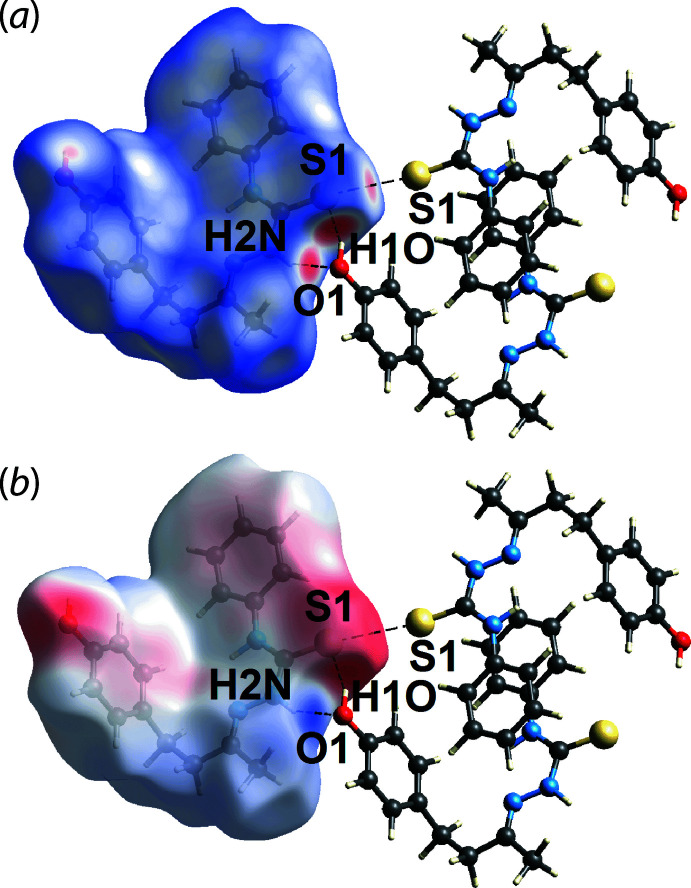
Views of the Hirshfeld surface for (I) mapped over (*a*) *d*_norm_ in the range −0.490 to +1.188 arbitrary units and (*b*) the calculated electrostatic potential in the range of −0.072 to +0.133 atomic units.

**Figure 4:**
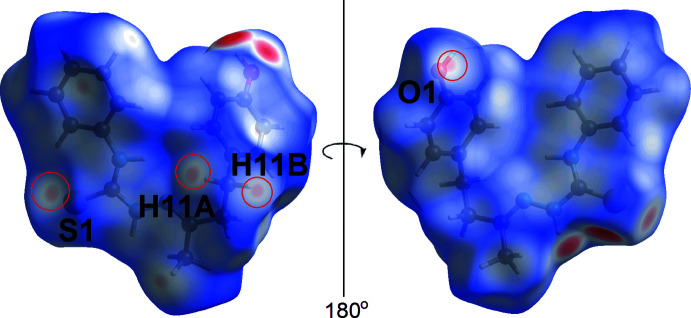
Two views of the Hirshfeld surface mapped for (I) over (*a*) *d*_norm_ in the range −0.490 to +1.188 arbitrary units.

**Figure 5:**
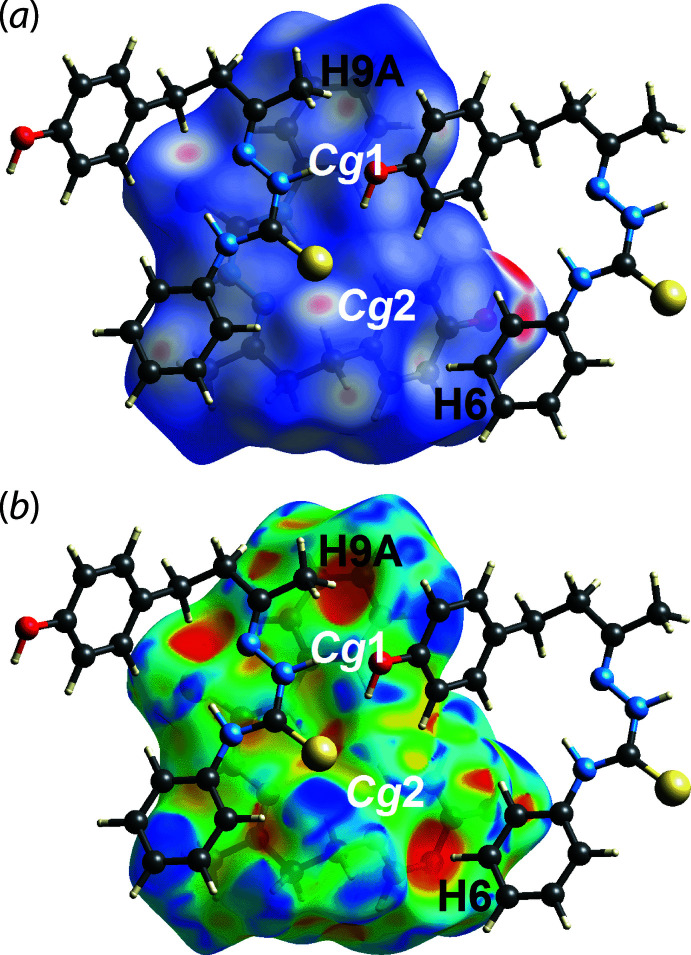
Views of the Hirshfeld surface mapped for (I) over (*a*) *d*_norm_ in the range −0.490 to +1.188 arbitrary units and (*b*) the shape-index property, each highlighting the methyl-C9—H9*A*⋯π(C2–C7; *Cg*1) and phenyl-C6—H6⋯π(C12–C17; *Cg*2) inter­actions.

**Figure 6:**
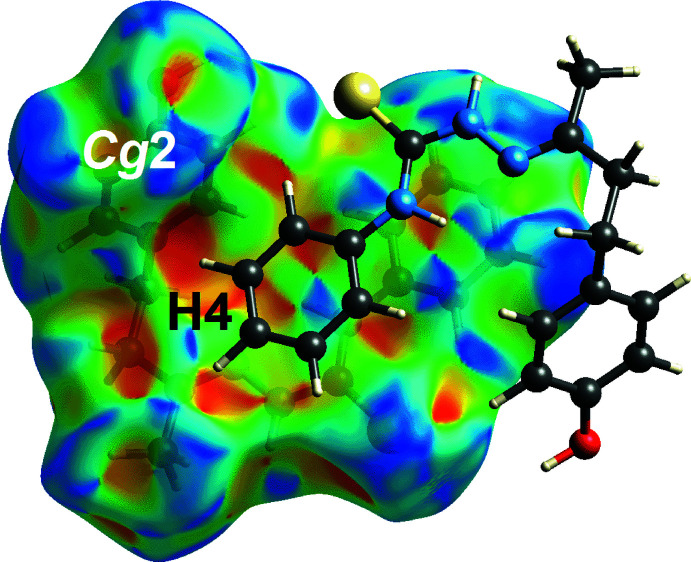
A view of the Hirshfeld surface mapped for (I) over the shape-index property highlighting phenyl-C4—H4⋯π(C12–C17; *Cg*2) inter­action.

The bright-red spots on the Hirshfeld surface mapped over *d*_norm_ in Fig. 3 ▸(*a*), *i.e*. near the amine-H2*N* and thione-S1 atoms, correspond to the amine-N2—H2*N*⋯O1(hydrox­yl), hydroxyl-O1—H1*O*⋯S1(thione) hydrogen bonds and the thione-S1⋯S1(thione) short contact; these and other short contacts calculated using *Crystal Explorer 17* are collated in Table 2 ▸. These hydrogen bonds are also reflected in the Hirshfeld surface mapped over the electrostatic potential shown in Fig. 3 ▸(*b*), where the positive electrostatic potential (blue) and negative electrostatic potential (red) regions are observed around the amine-H2*N* and thione-S1 atoms, respectively. The faint red spots appearing near the thione-S1, hydroxyl-O1 and methyl­ene-H11*A* and H11*B* atoms (Fig. 4 ▸) correspond to methyl­ene-C—H⋯S(thione) and methyl­ene-C—H⋯O1(hydrox­yl) inter­actions, with separations ∼0.2 Å shorter than the sum of their respective van der Waals radii, Table 2 ▸. The methyl-C9—H9*A*⋯π(C2–C7; *Cg*1) and phenyl-C6—H6⋯π(C12–C17; *Cg*2) inter­actions are shown as faint red spots on the *d*_norm_ surface in Fig. 5 ▸(*a*) and as two distinctive orange ‘potholes’ on the shape-index-mapped over Hirshfeld surface in Fig. 5 ▸(*b*). It is noted that the phenyl-C4—H4⋯π(C12–C17, *Cg*2) inter­action, Table 1 ▸, was not manifested on the *d*_norm_-mapped Hirshfeld surface. However, this inter­action clearly shows up as an orange ‘pothole’ on the shape-index-mapped Hirshfeld surface in Fig. 6 ▸.

**Figure 7:**
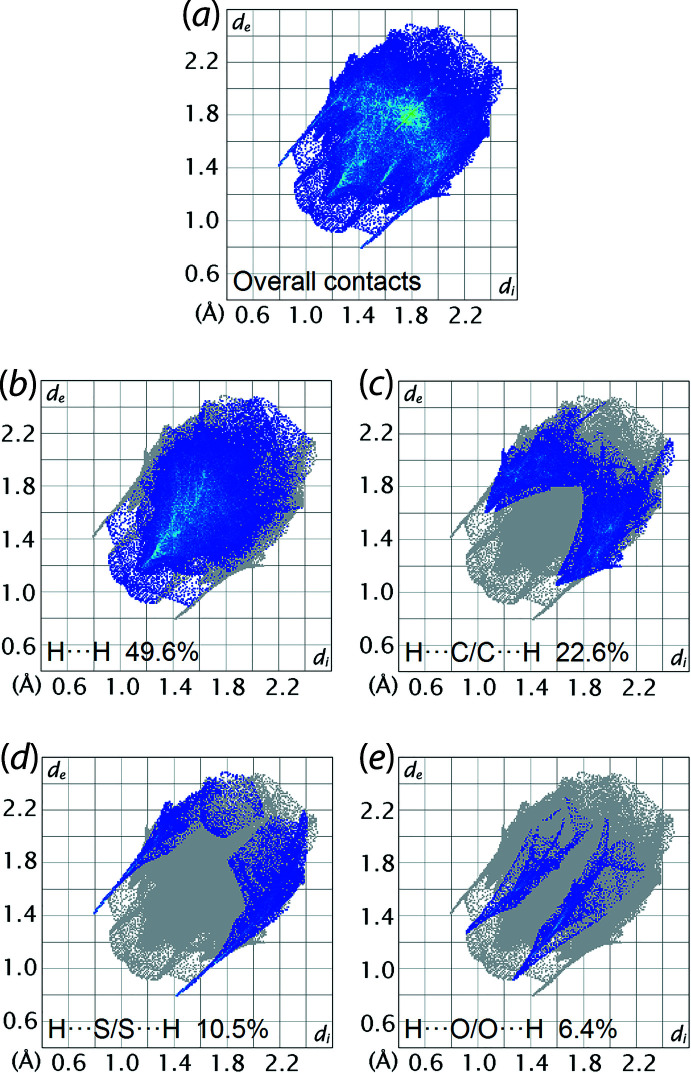
(*a*) A comparison of the full two-dimensional fingerprint plot for (I) and those delineated into (*b*) H⋯H, (*c*) H⋯C/C⋯H, (*d*) H⋯S/S⋯H and (*e*) H⋯O/O⋯H contacts.

The overall two-dimensional fingerprint plot computed for (I) is shown in Fig. 7 ▸(*a*) and those delineated into H⋯H, H⋯C/C⋯H, H⋯S/S⋯H and H⋯O/O⋯H contacts are illustrated in Fig. 7 ▸(*b*)–(*e*), respectively. The percentage contributions to the Hirshfeld surface of (I) from the different inter­atomic contacts are summarized in Table 3 ▸. The H⋯H contacts are the most prominent of all contacts and contribute 49.6% to the entire surface. The H⋯H contact manifested as a duckbill peak tipped at *d*_e_ = *d*_i_ ∼2.1 Å, Fig. 7 ▸(*b*), corresponds to the intra-layer H1*O*⋯H2*N* contact listed in Table 2 ▸. The H⋯C/C⋯H contacts contribute 22.6% to the Hirshfeld surface, Fig. 7 ▸(*c*), reflecting the significant C—H⋯π inter­actions evinced in the packing analysis, Table 1 ▸. Consistent with the O—H⋯S and N—H⋯O hydrogen bonds occurring in the crystal, H⋯S/S⋯H and H⋯O/O⋯H contacts contribute 10.5 and 6.4%, respectively, to the overall Hirshfeld surface. These two contacts appear as two sharp spikes in the fingerprint plots at *d*_e_ = *d*_i_ ≃ 2.2 Å in Fig. 7 ▸(*d*) and (*e*), respectively. The contributions from the other six inter­atomic contacts summarized in Table 3 ▸ have a reduced influence on the calculated Hirshfeld surface of (I), as each contributes less than 3.0%.

## Computational chemistry   

The energy frameworks were calculated for (I) by summing the four energy components – the electrostatic (*E*_ele_), polarization (*E*_pol_), dispersion (*E*_dis_) and exchange-repulsion (*E*_rep_) energy components (Turner *et al.*, 2017 ▸). The individual energy components as well as the total energy for the identified inter­molecular inter­actions are summarized in Table 4 ▸. As the intra-layer region is mainly consolidated by C—H⋯π and C—H⋯S/O inter­actions, the *E*_dis_ component makes the major contribution to the inter­action energies. The most significant stabilization energies are found in the intra-layer region, as outlined in *Supra­molecular Features*. The S1⋯S1 short contact is dominated by the *E*_ele_ (−8.9 kJ mol^−1^) and *E*_rep_ (12.2 kJ mol^−1^) terms and having a total energy of 11.7 kJ mol^−1^ is non-attractive.

The stabilization energies in the inter-layer region are dominated by the *E*_dis_ component. The greatest stabilization energy in the inter-layer region arises from methyl-C9—H9*B*⋯O1(hydrox­yl) [2.87 Å; −*x* + 1, −*y* + 2, −*z*] and methyl­ene-H10⋯H16(hy­droxy­benzene) inter­actions [2.59 Å; −*x* + 1, −*y* + 2, −*z*], which sum to −30.7 kJ mol^−1^. Generally, the long-range H⋯H contacts are the major inter­actions stabilizing the mol­ecules within the inter-layer region.

**Figure 8:**
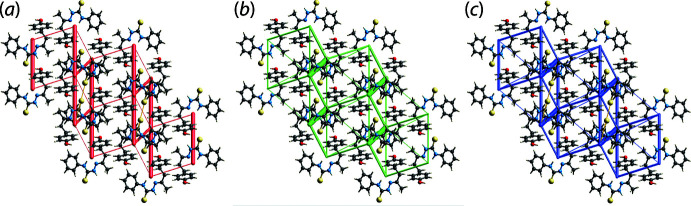
Perspective views of the energy frameworks calculated for (I) showing (*a*) electrostatic potential force, (*b*) dispersion force and (*c*) total energy, each plotted down the *a* axis. The radii of the cylinders are proportional to the relative magnitudes of the corresponding energies and were adjusted to the same scale factor of 55 with a cut-off value of 5 kJ mol^−1^.

Views of the energy framework diagrams down the *a* axis direction are shown in Fig. 8 ▸ and serve to emphasize the contribution of dispersion forces to the overall mol­ecular packing. The total *E*_ele_ of all pairwise inter­actions sum to −145.4 kJ mol^−1^, while the *E*_dis_ totals −342.1 kJ mol^−1^.

## Database survey   

In the crystallographic literature, there are two precedents for mol­ecules related to (I) in which the imine bond is connected to an aromatic residue *via* an ethane link. Each of these is a *N*-methyl species, *i.e*. MeN(H)C(=S)N(H)N=C(Me)CH_2_CH_2_Ar, one with Ar = phenyl (Tan, Kwong *et al.*, 2020*a* ▸) and the other with Ar = 4-meth­oxy­benzene (Tan *et al.*, 2012 ▸). In the former, the mol­ecule has a distinctive U-shaped conformation with a twist about the CH_2_—CH_2_ bond [the C_i_—C_m_—C_m_—C_p_ (i = imine, m = methyl­ene, p = phen­yl) torsion angle = −62.76 (16)°], a conformation stabilized, at least in part, by an intra­molecular amine-N—H⋯π(phen­yl) inter­action. By contrast, in the species with Ar = 4-meth­oxy­benzene, the mol­ecule is close to planar as indicated by the C_i_—C_m_—C_m_—C_p_ torsion angles of 177.51 (12) and −175.80 (12)°, respectively, for the two independent molecules comprising the asymmetric unit. Thus, to a first approximation, the conformation observed in (I) matches that seen in the species with Ar = phenyl, even though no intra­molecular N—H⋯π(hy­droxy­benzene) inter­action was noted in (I).

## Synthesis and crystallization   

4-Phenyl-3-thio­semicarbazide (10 mmol) dissolved in hot absolute ethanol (50 ml) was combined with 4-(4-hy­droxy­phen­yl)-2-butanone (10 mmol), dissolved in hot absolute ethanol (50 ml) with a few drops of concentrated hydro­chloric acid added as catalyst. The mixture was heated (348 K) and stirred for about 30 min. The mixture was allowed to cool to room temperature while stirring. The white precipitate was filtered, washed with cold ethanol and dried *in vacuo*. Single crystals were grown at room temperature in mixed solvents of dimethyformamide and aceto­nitrile (1:2 *v*/*v*) by slow evaporation. ^1^H NMR (500 MHz, CDCl_3_, referenced to TMS): δ 9.14 (*s*, 1H), 8.59 (*s*, 1H), 7.59 (*d*, 2H), 7.37 (*t*, 2H), 7.22 (*t*, 1H), 7.03 (*d*, 2H), 6.76 (*d*, 2H), 5.46 (*s*, 1H), 2.83 (*t*, 2H), 2.61 (*t*, 2H), 1.90 (*s*, 3H). ^13^C NMR (500 MHz, CDCl_3_, referenced to solvent, 77.16 ppm): δ 176.22, 154.16, 152.02, 137.93, 132.72, 129.39, 128.84, 126.16, 124.39, 115.57, 40.38, 31.58, 16.19.

## Refinement   

Crystal data, data collection and structure refinement details are summarized in Table 5 ▸. The carbon-bound H-atoms were placed in calculated positions (C—H = 0.95–0.99 Å) and were included in the refinement in the riding-model approximation, with *U*_iso_(H) set to 1.2–1.5*U*_eq_(C). The O-bound and N-bound H atoms were located in a difference-Fourier map but were refined with O—H = 0.84±0.01 and N—H = 0.88±0.01 Å distance restraints, respectively, and with *U*_iso_(H) set to 1.5*U*_eq_(O) and 1.2*U*_eq_(N).

## Supplementary Material

Crystal structure: contains datablock(s) I, global. DOI: 10.1107/S2056989021006666/hb7978sup1.cif

Structure factors: contains datablock(s) I. DOI: 10.1107/S2056989021006666/hb7978Isup2.hkl

Supporting information file. DOI: 10.1107/S2056989021006666/hb7978Isup3.cml

CCDC reference: 2092413

Additional supporting information:  crystallographic information; 3D view; checkCIF report

## Figures and Tables

**Table 1:** Hydrogen-bond geometry (Å, °) *Cg*1 and *Cg*2 are the centroids of the (C2–C7) and (C12–C17) rings, respectively.

*D*—H⋯*A*	*D*—H	H⋯*A*	*D*⋯*A*	*D*—H⋯*A*
N1—H1*N*⋯N3	0.88 (2)	2.10 (2)	2.6214 (19)	117 (1)
O1—H1*O*⋯S1^i^	0.84 (1)	2.34 (2)	3.1489 (13)	162 (2)
N2—H2*N*⋯O1^ii^	0.87 (2)	2.31 (2)	3.1219 (19)	155 (2)
C11—H11*A*⋯S1^iii^	0.99	2.84	3.7936 (17)	163
C11—H11*B*⋯O1^iv^	0.99	2.58	3.438 (2)	145
C9—H9*A*⋯*Cg*1^iii^	0.98	2.90	3.6862 (19)	138
C4—H4⋯*Cg*2^v^	0.95	2.90	3.6939 (19)	142
C6—H6⋯*Cg*2^vi^	0.98	2.84	3.601 (2)	138

Symmetry codes: (i) x-1, y+1, z; (ii) x+1, y-1, z; (iii) -x+2, -y+1, -z+1; (iv) x+1, y, z; (v) -x+1, -y+1, -z+1; (vi) -x+1, -y+2, -z+1.

**Table 2:** A summary of short inter­atomic contacts (Å) for (I)*^*a*^*

Contact	Distance	Symmetry operation
O1—H1*O*⋯S1*^*b*^*	2.20	*x* − 1, *y* + 1, *z*
N2—H2*N*⋯O1*^*b*^*	2.19	*x* + 1, *y* − 1, *z*
S1⋯S1	3.35	−*x* + 2, −*y* + 1, −*z* + 1
C11—H11*A*⋯S1	2.75	−*x* + 2, −*y* + 1, −*z* + 1
C11—H11*B*⋯O1	2.50	*x* + 1, *y*, *z*
C9—H9*A*⋯*Cg*(C2–C7)	2.90	−*x* + 2, −*y* + 1, −*z* + 1
C6—H6⋯*Cg*(C12–C17)	2.84	−*x* + 1, −*y* + 2, −*z* + 1
C4—H4⋯*Cg*(C12–C17)	2.90	−*x* + 1, −*y* + 1, −*z* + 1
H1*O*⋯H2*N*	2.05	*x* − 1, *y* + 1, *z*

Notes: (*a*) The inter­atomic distances are calculated in *Crystal Explorer 17* (Turner *et al.*, 2017 ▸) with the *X*—H bond lengths adjusted to their neutron values. (*b*) This contact corresponds to a conventional hydrogen bond.

**Table 3:** The percentage contributions from inter­atomic contacts to the Hirshfeld surface for (I)

Contact	Percentage contribution
H⋯H	49.6
H⋯C/C⋯H	22.6
H⋯S/S⋯H	10.5
H⋯O/O⋯H	6.4
C⋯C	2.9
N⋯C/C⋯N	2.9
H⋯N/N⋯H	2.8
N⋯N	1.0
S⋯S	0.8
S⋯C/C⋯S	0.5

**Table 4:** A summary of inter­action energies (kJ mol^−1^) calculated for (I)

Contact	*R* (Å)	*E*_ele_	*E*_pol_	*E*_dis_	*E*_rep_	*E*_tot_
Intra-layer region						
C11—H11*A*⋯S1^iii^ +						
C9—H9*A*⋯*Cg*(C2—C7)^v^	5.43	−38.9	−11.6	−84.9	76.0	−76.7
C4—H4⋯*Cg*(C12—C17)^vi^	5.30	−26.8	−7.3	−95.7	66.8	−75.8
O1—H1*O*⋯S1^i^ +						
N2—H2*N*⋯O1^ii^	10.02	−64.6	−14.9	−21.3	84.4	−45.7
C6—H6⋯*Cg*(C12—C17)^vii^	7.12	−5.8	−2.1	−44.2	30.4	−27.4
C11—H11*B*⋯O1^iv^	8.06	−2.2	−1.0	−9.9	6.5	−7.7
C6⋯H1*O* ^viii^	11.11	−0.3	−0.4	−3.5	0.0	−3.6
S1⋯S1^ix^	11.56	8.9	−1.8	−4.4	12.2	11.7
Inter-layer region						
C9—H9*B*⋯O1^*x*^ +						
H10*A*⋯H16^*x*^	8.90	−11.2	−2.7	−28.8	13.3	−30.7
H10*A*⋯H10*B* ^xi^ +						
H10*B*⋯H17^xi^	10.63	0.8	−1.8	−24.9	17.1	−11.6
H4⋯H17^xii^ +						
H5⋯H16^xii^	12.23	−4.0	−0.5	−11.1	6.6	−10.2
H9*C*⋯H9*C* ^xiii^	10.35	−2.8	−1.4	−9.5	7.3	−7.8
H5⋯H9*B* ^xiv^	13.13	1.5	−0.3	−3.9	1.2	−1.4

Symmetry codes: (i) *x* − 1, *y* + 1, *z*; (ii) *x* + 1, *y* − 1, *z*; (iii) −*x* + 2, −*y* + 1, −*z* + 1; (iv) *x* + 1, *y*, *z*; (v) −*x* + 2, −*y* + 1, −*z* + 1; (vi) −*x* + 1, −*y* + 1, −*z* + 1; (vii) −*x* + 1, −*y* + 2, −*z* + 1; (viii) −*x*, −*y* + 2, −*z* + 1; (ix) −*x* + 2, −*y*, −*z* + 1; (*x*) −*x* + 1, −*y* + 2, −*z*; (xi) −*x* + 2, −*y* + 2, −*z*; (xii) *x*, *y* − 1, *z* + 1; (xiii) −*x* + 2, −*y*, −*z* + 1; (xiv) *x* − 1, *y*, *z* + 1.

**Table 5:** Experimental details

Crystal data	
Chemical formula	C_17_H_19_N_3_OS
*M*_r_	313.41
Crystal system, space group	Triclinic, *P*\overline{1}
Temperature (K)	100
*a*, *b*, *c* (Å)	8.0605 (6), 9.5635 (6), 11.4397 (6)
α, β, γ (°)	70.578 (5), 82.671 (5), 68.723 (6)
*V* (Å^3^)	774.95 (9)
*Z*	2
Radiation type	Cu *K*α
μ (mm^−1^)	1.89
Crystal size (mm)	0.35 × 0.21 × 0.04

Data collection	
Diffractometer	Oxford Diffraction Gemini
Absorption correction	Multi-scan (*CrysAlis PRO*; Agilent, 2012 ▸)
*T*_min_, *T*_max_	0.52, 0.93
No. of measured, independent and observed [*I* > 2σ(*I*)] reflections	15291, 2982, 2712
*R*_int_	0.032
(sin θ/λ)_max_ (Å^−1^)	0.616

Refinement	
*R*[*F* ^2^ > 2σ(*F* ^2^)], *wR*(*F* ^2^), *S*	0.039, 0.109, 1.03
No. of reflections	2982
No. of parameters	209
No. of restraints	3
H-atom treatment	H atoms treated by a mixture of independent and constrained refinement
Δρ_max_, Δρ_min_ (e Å^−3^)	0.36, −0.21

Computer programs: *CrysAlis PRO* (Agilent, 2012 ▸), *SHELXT* (Sheldrick, 2015*a* ▸), *SHELXL2018/3* (Sheldrick, 2015*b* ▸), *ORTEP-3 for Windows* (Farrugia, 2012 ▸), *DIAMOND* (Brandenburg, 2006 ▸) and *publCIF* (Westrip, 2010 ▸).

## supplementary crystallographic information

### Crystal data

**Table:**

C_17_H_19_N_3_OS	*Z* = 2
*M**_r_* = 313.41	*F*(000) = 332
Triclinic, *P*1	*D*_x_ = 1.343 Mg m^−^^3^
*a* = 8.0605 (6) Å	Cu *K*α radiation, λ = 1.5418 Å
*b* = 9.5635 (6) Å	Cell parameters from 7107 reflections
*c* = 11.4397 (6) Å	θ = 4–72°
α = 70.578 (5)°	µ = 1.89 mm^−^^1^
β = 82.671 (5)°	*T* = 100 K
γ = 68.723 (6)°	Plate, colourless
*V* = 774.95 (9) Å^3^	0.35 × 0.21 × 0.04 mm

### Data collection

**Table:**

Oxford Diffraction Gemini diffractometer	2712 reflections with *I* > 2σ(*I*)
Graphite monochromator	*R*_int_ = 0.032
ω scans	θ_max_ = 71.9°, θ_min_ = 4.1°
Absorption correction: multi-scan (CrysAlisPro; Agilent, 2012)	*h* = −9→9
*T*_min_ = 0.52, *T*_max_ = 0.93	*k* = −11→11
15291 measured reflections	*l* = −13→13
2982 independent reflections	

### Refinement

**Table:**

Refinement on *F*^2^	Primary atom site location: structure-invariant direct methods
Least-squares matrix: full	Secondary atom site location: difference Fourier map
*R*[*F*^2^ > 2σ(*F*^2^)] = 0.039	Hydrogen site location: mixed
*wR*(*F*^2^) = 0.109	H atoms treated by a mixture of independent and constrained refinement
*S* = 1.03	*w* = 1/[σ^2^(*F*_o_^2^) + (0.0658*P*)^2^ + 0.2814*P*] where *P* = (*F*_o_^2^ + 2*F*_c_^2^)/3
2982 reflections	(Δ/σ)_max_ < 0.001
209 parameters	Δρ_max_ = 0.36 e Å^−^^3^
3 restraints	Δρ_min_ = −0.21 e Å^−^^3^

### Special details

**Table:**

Geometry. All esds (except the esd in the dihedral angle between two l.s. planes) are estimated using the full covariance matrix. The cell esds are taken into account individually in the estimation of esds in distances, angles and torsion angles; correlations between esds in cell parameters are only used when they are defined by crystal symmetry. An approximate (isotropic) treatment of cell esds is used for estimating esds involving l.s. planes.

### Fractional atomic coordinates and isotropic or equivalent isotropic displacement parameters (Å^2^)

**Table:**

	*x*	*y*	*z*	*U*_iso_*/*U*_eq_	
S1	0.93080 (5)	0.19624 (4)	0.46989 (4)	0.02796 (15)	
O1	0.12066 (15)	1.17646 (14)	0.21473 (11)	0.0292 (3)	
H1O	0.082 (3)	1.159 (3)	0.2882 (11)	0.044*	
N1	0.77812 (17)	0.49550 (15)	0.48469 (12)	0.0233 (3)	
H1N	0.774 (2)	0.5919 (13)	0.4427 (16)	0.028*	
N2	0.95702 (17)	0.46117 (15)	0.31940 (12)	0.0237 (3)	
H2N	1.024 (2)	0.3997 (19)	0.2772 (15)	0.028*	
N3	0.92493 (17)	0.62221 (15)	0.27822 (12)	0.0235 (3)	
C1	0.8821 (2)	0.39389 (18)	0.42601 (14)	0.0223 (3)	
C2	0.6701 (2)	0.47628 (18)	0.59294 (14)	0.0219 (3)	
C3	0.6397 (2)	0.33626 (18)	0.66028 (15)	0.0247 (3)	
H3	0.695700	0.243795	0.636170	0.030*	
C4	0.5271 (2)	0.33353 (19)	0.76263 (15)	0.0254 (3)	
H4	0.506601	0.238203	0.808407	0.030*	
C5	0.4437 (2)	0.4669 (2)	0.79964 (15)	0.0268 (4)	
H5	0.366970	0.463257	0.870039	0.032*	
C6	0.4742 (2)	0.6063 (2)	0.73207 (16)	0.0300 (4)	
H6	0.417842	0.698703	0.756202	0.036*	
C7	0.5864 (2)	0.61034 (19)	0.62989 (15)	0.0271 (4)	
H7	0.606583	0.705860	0.584298	0.033*	
C8	0.9943 (2)	0.67483 (18)	0.17277 (15)	0.0241 (3)	
C9	1.1028 (2)	0.5771 (2)	0.09267 (16)	0.0323 (4)	
H9A	1.224584	0.522396	0.124235	0.048*	
H9B	1.104985	0.645704	0.007433	0.048*	
H9C	1.049551	0.499250	0.094012	0.048*	
C10	0.9738 (2)	0.84652 (19)	0.12493 (15)	0.0265 (4)	
H10A	0.909132	0.893089	0.045447	0.032*	
H10B	1.094242	0.854018	0.106361	0.032*	
C11	0.8784 (2)	0.94844 (19)	0.20771 (15)	0.0269 (4)	
H11A	0.922435	0.889481	0.293362	0.032*	
H11B	0.911704	1.044499	0.179824	0.032*	
C12	0.6776 (2)	0.99862 (17)	0.21054 (15)	0.0234 (3)	
C13	0.5843 (2)	0.97100 (18)	0.32206 (15)	0.0254 (3)	
H13	0.648932	0.912561	0.396999	0.030*	
C14	0.3991 (2)	1.02660 (18)	0.32684 (15)	0.0260 (3)	
H14	0.338550	1.005371	0.404055	0.031*	
C15	0.3032 (2)	1.11362 (18)	0.21756 (15)	0.0247 (3)	
C16	0.3935 (2)	1.14113 (18)	0.10509 (15)	0.0257 (3)	
H16	0.328803	1.198894	0.030121	0.031*	
C17	0.5781 (2)	1.08424 (18)	0.10229 (15)	0.0253 (3)	
H17	0.638311	1.103991	0.024812	0.030*	

### Atomic displacement parameters (Å^2^)

**Table:**

	*U*^11^	*U*^22^	*U*^33^	*U*^12^	*U*^13^	*U*^23^
S1	0.0328 (2)	0.0182 (2)	0.0289 (2)	−0.00720 (17)	0.00688 (16)	−0.00644 (16)
O1	0.0263 (6)	0.0318 (6)	0.0311 (6)	−0.0113 (5)	0.0057 (5)	−0.0126 (5)
N1	0.0245 (7)	0.0172 (6)	0.0265 (7)	−0.0084 (5)	0.0031 (5)	−0.0044 (5)
N2	0.0239 (7)	0.0194 (6)	0.0260 (7)	−0.0073 (5)	0.0050 (5)	−0.0067 (5)
N3	0.0212 (6)	0.0201 (6)	0.0280 (7)	−0.0085 (5)	0.0002 (5)	−0.0044 (5)
C1	0.0192 (7)	0.0218 (8)	0.0252 (8)	−0.0069 (6)	−0.0010 (6)	−0.0064 (6)
C2	0.0190 (7)	0.0232 (8)	0.0231 (7)	−0.0072 (6)	−0.0002 (6)	−0.0067 (6)
C3	0.0243 (8)	0.0222 (8)	0.0287 (8)	−0.0088 (6)	0.0013 (6)	−0.0086 (6)
C4	0.0253 (8)	0.0247 (8)	0.0260 (8)	−0.0116 (7)	0.0008 (6)	−0.0046 (6)
C5	0.0256 (8)	0.0302 (8)	0.0245 (8)	−0.0105 (7)	0.0045 (6)	−0.0090 (7)
C6	0.0338 (9)	0.0255 (8)	0.0311 (9)	−0.0089 (7)	0.0041 (7)	−0.0124 (7)
C7	0.0326 (9)	0.0213 (8)	0.0273 (8)	−0.0106 (7)	0.0028 (7)	−0.0069 (6)
C8	0.0177 (7)	0.0250 (8)	0.0266 (8)	−0.0068 (6)	−0.0010 (6)	−0.0045 (6)
C9	0.0331 (9)	0.0321 (9)	0.0275 (9)	−0.0096 (7)	0.0034 (7)	−0.0073 (7)
C10	0.0222 (8)	0.0257 (8)	0.0284 (8)	−0.0110 (6)	0.0025 (6)	−0.0021 (7)
C11	0.0291 (9)	0.0238 (8)	0.0291 (8)	−0.0140 (7)	−0.0023 (7)	−0.0037 (6)
C12	0.0287 (8)	0.0174 (7)	0.0270 (8)	−0.0116 (6)	0.0008 (6)	−0.0069 (6)
C13	0.0342 (9)	0.0193 (7)	0.0238 (8)	−0.0115 (6)	−0.0017 (6)	−0.0051 (6)
C14	0.0352 (9)	0.0218 (8)	0.0250 (8)	−0.0143 (7)	0.0076 (7)	−0.0100 (6)
C15	0.0270 (8)	0.0205 (7)	0.0307 (8)	−0.0114 (6)	0.0045 (6)	−0.0113 (6)
C16	0.0294 (8)	0.0239 (8)	0.0243 (8)	−0.0096 (7)	−0.0002 (6)	−0.0076 (6)
C17	0.0295 (8)	0.0240 (8)	0.0240 (8)	−0.0120 (6)	0.0038 (6)	−0.0078 (6)

### Geometric parameters (Å, º)

**Table:**

S1—C1	1.6910 (15)	C8—C9	1.500 (2)
O1—C15	1.373 (2)	C8—C10	1.501 (2)
O1—H1O	0.842 (10)	C9—H9A	0.9800
N1—C1	1.340 (2)	C9—H9B	0.9800
N1—C2	1.419 (2)	C9—H9C	0.9800
N1—H1N	0.877 (9)	C10—C11	1.524 (2)
N2—C1	1.356 (2)	C10—H10A	0.9900
N2—N3	1.3857 (18)	C10—H10B	0.9900
N2—H2N	0.874 (9)	C11—C12	1.512 (2)
N3—C8	1.284 (2)	C11—H11A	0.9900
C2—C7	1.392 (2)	C11—H11B	0.9900
C2—C3	1.397 (2)	C12—C13	1.392 (2)
C3—C4	1.386 (2)	C12—C17	1.396 (2)
C3—H3	0.9500	C13—C14	1.392 (2)
C4—C5	1.387 (2)	C13—H13	0.9500
C4—H4	0.9500	C14—C15	1.393 (2)
C5—C6	1.392 (2)	C14—H14	0.9500
C5—H5	0.9500	C15—C16	1.389 (2)
C6—C7	1.382 (2)	C16—C17	1.387 (2)
C6—H6	0.9500	C16—H16	0.9500
C7—H7	0.9500	C17—H17	0.9500
			
C15—O1—H1O	108.4 (15)	H9A—C9—H9B	109.5
C1—N1—C2	132.42 (13)	C8—C9—H9C	109.5
C1—N1—H1N	110.3 (13)	H9A—C9—H9C	109.5
C2—N1—H1N	117.2 (13)	H9B—C9—H9C	109.5
C1—N2—N3	120.90 (13)	C8—C10—C11	117.78 (14)
C1—N2—H2N	117.6 (13)	C8—C10—H10A	107.9
N3—N2—H2N	121.4 (13)	C11—C10—H10A	107.9
C8—N3—N2	115.76 (14)	C8—C10—H10B	107.9
N1—C1—N2	114.40 (13)	C11—C10—H10B	107.9
N1—C1—S1	128.00 (12)	H10A—C10—H10B	107.2
N2—C1—S1	117.60 (12)	C12—C11—C10	115.73 (13)
C7—C2—C3	119.28 (15)	C12—C11—H11A	108.3
C7—C2—N1	115.94 (13)	C10—C11—H11A	108.3
C3—C2—N1	124.75 (15)	C12—C11—H11B	108.3
C4—C3—C2	119.36 (15)	C10—C11—H11B	108.3
C4—C3—H3	120.3	H11A—C11—H11B	107.4
C2—C3—H3	120.3	C13—C12—C17	117.43 (15)
C3—C4—C5	121.43 (14)	C13—C12—C11	121.19 (14)
C3—C4—H4	119.3	C17—C12—C11	121.22 (14)
C5—C4—H4	119.3	C14—C13—C12	121.84 (15)
C4—C5—C6	119.00 (15)	C14—C13—H13	119.1
C4—C5—H5	120.5	C12—C13—H13	119.1
C6—C5—H5	120.5	C13—C14—C15	119.51 (15)
C7—C6—C5	120.06 (16)	C13—C14—H14	120.2
C7—C6—H6	120.0	C15—C14—H14	120.2
C5—C6—H6	120.0	O1—C15—C16	117.28 (14)
C6—C7—C2	120.87 (15)	O1—C15—C14	123.09 (15)
C6—C7—H7	119.6	C16—C15—C14	119.62 (15)
C2—C7—H7	119.6	C17—C16—C15	119.96 (15)
N3—C8—C9	125.38 (14)	C17—C16—H16	120.0
N3—C8—C10	118.97 (15)	C15—C16—H16	120.0
C9—C8—C10	115.60 (14)	C16—C17—C12	121.62 (15)
C8—C9—H9A	109.5	C16—C17—H17	119.2
C8—C9—H9B	109.5	C12—C17—H17	119.2
			
C1—N2—N3—C8	−176.20 (13)	N2—N3—C8—C10	−176.77 (12)
C2—N1—C1—N2	176.34 (14)	N3—C8—C10—C11	2.8 (2)
C2—N1—C1—S1	−4.5 (2)	C9—C8—C10—C11	−174.92 (13)
N3—N2—C1—N1	0.0 (2)	C8—C10—C11—C12	−78.12 (18)
N3—N2—C1—S1	−179.27 (11)	C10—C11—C12—C13	126.58 (16)
C1—N1—C2—C7	176.90 (15)	C10—C11—C12—C17	−58.04 (19)
C1—N1—C2—C3	−5.1 (3)	C17—C12—C13—C14	−0.3 (2)
C7—C2—C3—C4	−0.1 (2)	C11—C12—C13—C14	175.22 (14)
N1—C2—C3—C4	−178.09 (14)	C12—C13—C14—C15	−0.6 (2)
C2—C3—C4—C5	0.1 (2)	C13—C14—C15—O1	−178.06 (14)
C3—C4—C5—C6	0.1 (2)	C13—C14—C15—C16	1.3 (2)
C4—C5—C6—C7	−0.1 (2)	O1—C15—C16—C17	178.31 (13)
C5—C6—C7—C2	0.0 (3)	C14—C15—C16—C17	−1.0 (2)
C3—C2—C7—C6	0.1 (2)	C15—C16—C17—C12	0.1 (2)
N1—C2—C7—C6	178.22 (14)	C13—C12—C17—C16	0.5 (2)
N2—N3—C8—C9	0.7 (2)	C11—C12—C17—C16	−175.00 (14)

### Hydrogen-bond geometry (Å, º)

*Cg*1 and *Cg*2 are the centroids of the (C2–C7) and (C12–C17) rings, respectively.

**Table:**

*D*—H···*A*	*D*—H	H···*A*	*D*···*A*	*D*—H···*A*
N1—H1*N*···N3	0.88 (2)	2.10 (2)	2.6214 (19)	117 (1)
O1—H1*O*···S1^i^	0.84 (1)	2.34 (2)	3.1489 (13)	162 (2)
N2—H2*N*···O1^ii^	0.87 (2)	2.31 (2)	3.1219 (19)	155 (2)
C11—H11*A*···S1^iii^	0.99	2.84	3.7936 (17)	163
C11—H11*B*···O1^iv^	0.99	2.58	3.438 (2)	145
C9—H9*A*···*Cg*1^iii^	0.98	2.90	3.6862 (19)	138
C4—H4···*Cg*2^v^	0.95	2.90	3.6939 (19)	142
C6—H6···*Cg*2^vi^	0.98	2.84	3.601 (2)	138

Symmetry codes: (i) *x*−1, *y*+1, *z*; (ii) *x*+1, *y*−1, *z*; (iii) −*x*+2, −*y*+1, −*z*+1; (iv) *x*+1, *y*, *z*; (v) −*x*+1, −*y*+1, −*z*+1; (vi) −*x*+1, −*y*+2, −*z*+1.
